# Experimental infection of pregnant goats with bovine viral diarrhea virus (BVDV) 1 or 2

**DOI:** 10.1186/1297-9716-45-38

**Published:** 2014-04-04

**Authors:** Thomas Passler, Kay P Riddell, Misty A Edmondson, Manuel F Chamorro, John D Neill, Bruce W Brodersen, Heather L Walz, Patricia K Galik, Yijing Zhang, Paul H Walz

**Affiliations:** 1 College of Veterinary Medicine, Departments of Clinical Sciences and Pathobiology, Auburn University, Auburn, AL 36849, USA; 2 Ruminant Diseases and Immunology Research Unit, National Animal Disease Center, USDA, ARS, Ames, IA 50010, USA; 3 School of Veterinary Medicine and Biomedical Sciences, University of Nebraska, Lincoln, NE 68508, USA

## Abstract

Infections with bovine viral diarrhea virus (BVDV) of the genus pestivirus, family *Flaviviridae*, are not limited to cattle but occur in various artiodactyls. Persistently infected (PI) cattle are the main source of BVDV. Persistent infections also occur in heterologous hosts such as sheep and deer. BVDV infections of goats commonly result in reproductive disease, but viable PI goats are rare. Using 2 BVDV isolates, previously demonstrated to cause PI cattle and white-tailed deer, this study evaluated the outcome of experimental infection of pregnant goats. Pregnant goats (5 goats/group) were intranasally inoculated with BVDV 1b AU526 (group 1) or BVDV 2 PA131 (group 2) at approximately 25–35 days of gestation. The outcome of infection varied considerably between groups. In group 1, only 3 does became viremic, and 1 doe gave birth to a stillborn fetus and a viable PI kid, which appeared healthy and shed BVDV continuously. In group 2, all does became viremic, 4/5 does aborted, and 1 doe gave birth to a non-viable PI kid. Immunohistochemistry demonstrated BVDV antigen in tissues of evaluated fetuses, with similar distribution but reduced intensity as compared to cattle. The genetic sequence of inoculated viruses was compared to those from PI kids and their dam. Most nucleotide changes in group 1 were present during the dam’s acute infection. In group 2, a similar number of mutations resulted from fetal infection as from maternal acute infection. Results demonstrated that BVDV may cause reproductive disease but may also be maintained in goats.

## Introduction

Bovine viral diarrhea virus (BVDV) is the prototypic member of the genus pestivirus in the family *Flaviviridae*. The description of genetically distinct BVDV isolates from outbreaks of severe disease in North American cattle herds in the 1990’s prompted reclassification of BVDV into 2 species, BVDV 1 and BVDV 2 [1,2]. Infections with both species of BVDV can induce a wide spectrum of clinical manifestations with subtle to severe clinical signs resulting from respiratory, reproductive, or immunosuppressive diseases [3]. A central aspect in the maintenance and perpetuation of BVDV in cattle populations are persistently infected (PI) animals that are infected in utero prior to development of immunocompetence and shed BVDV for life.

Infections with BVDV are not limited to cattle but have been reported in various domestic and free-ranging artiodactyls. Evidence of BVDV infection exists in 7 of the 10 families comprising the mammalian order Artiodactyla including Antilocapridae, Bovidae, Camelidae, Cervidae, Giraffidae, Suidae, and Tragulidae, representing over 50 species [4]. As in cattle, clinical signs of BVDV infection in heterologous hosts are variable and depend on different host and virus-associated factors, but respiratory and reproductive diseases are commonly reported [4]. BVDV infection of heterologous hosts during pregnancy may manifest as reproductive failure and result in fetal resorption, fetal mummification, stillbirth, or abortion [4]. Congenital infection of the heterologous fetus may result in fetal death, fetal anomalies, developmental malformation of the fetal central nervous system, or birth of non-viable offspring [5-10]. An especially noteworthy outcome of congenital BVDV infection in heterologous hosts is the occurrence of persistent infection, which has been reported in different species [6,7,11-14]. The efficiency with which BVDV crosses the placental barrier and induces persistent infection appears to differ among species. In cattle, the rate of fetal infection and development of PI offspring following maternal BVDV infection during early pregnancy approaches 100% [15,16]. While efficient transplacental infection was also detected in white-tailed deer (*Odocoileus virginianus*), studies in domestic swine reported the occurrence of fetal infection in only 1 of 20 gilts or 1 of 43 fetuses born to 4 gilts, respectively [10,17,18].

BVDV infections of small ruminants are similar to those in cattle, and evidence of infection exists in many countries, where seroprevalence rates from 3 to 35% were detected [4]. BVDV seroprevalence rates are commonly greater than those for border disease virus [4]. In small ruminants, postnatal infections commonly cause mild clinical signs, including pyrexia and leucopenia [19]. Infections in pregnant small ruminants may result in uteroplacental pathology and pregnancy loss by fetal resorption or abortions [9]. In sheep, BVDV rapidly crosses the placental barrier, and the virus was detected in fetal tissues by RT-PCR and immunohistochemistry approximately 100 h following infection [20]. While transplacental BVDV infection in sheep can result in pregnancy losses and non-viability of lambs, reports of viable PI offspring are also common [6,21].

In goats, natural infections with both species of BVDV are reported [22,23]. Field outbreaks of BVDV-associated disease in goats are mainly characterized by pregnancy losses and neonatal morbidity and mortality [22,24]. Similarly, experimental inoculation of pregnant goats with cytopathic and non-cytopathic BVDV resulted in severe pregnancy losses with abortion and fetal death rates of up to 100% [9,25]. The occurrence of viable PI goats appears to be much rarer than in other species, such as cattle and sheep. However, two recent reports of viable PI goats born to dams infected during exposure to PI cattle infected with BVDV 1 exist [26,27].

The goal of the present study was to evaluate the outcome of experimental BVDV infection in pregnant goats at an earlier gestational age than in previous studies using BVDV 1 and 2 isolates that were previously documented to cause persistent infections in cattle and white-tailed deer [13,17].

## Materials and methods

### Experimental inoculation and sample schedule

The research described herein was performed under the approval of the Institutional Animal Care and Use Committee of Auburn University (2011–2014). All reported times are in reference to the time of BVDV inoculation (time 0). 10 adult, female, crossbred goats were obtained and confirmed to be negative for BVDV by virus isolation (VI) and BVDV-antibodies by virus neutralization (VN) using the BVDV strains BVDV 1b AU526 and BVDV 2 PA131. On day -38, following 10 days of fence-line contact, 2 adult bucks were placed into the same pasture to allow natural mating. Breeding success was evaluated by transrectal ultrasound examination on days: -9, -4, and -1 (29, 34, and 37 days following initial exposure to bucks). On day -1, all does appeared to be pregnant; however, in 3 does, pregnancy confirmation lacked confidence and the possibility of a false positive pregnancy confirmation was considered. In the remaining does, individual gestational lengths were estimated to be 25 to 35 days based on observations of breeding activity and ultrasound findings. Because of the desire to inoculate animals during early pregnancy, all does, including those without affirmed pregnancy status, remained in the study.

On day 0, does were randomly assigned to 1 of 2 groups and transported to 2 pastures at the North Auburn BVDV unit at Auburn University. Following collection of initial blood samples for VI and VN, 5 does per group were inoculated with either BVDV 1b or BVDV 2 of bovine origin. Using a disposable intranasal cannula designed to intranasally vaccinate calves, 10^5^ cell culture infective doses (50% endpoint) (CCID_50_) of the BVDV 1b AU526 (group 1) or BVDV 2 PA131 (group 2) strains diluted in 2 mL of minimum essential medium (MEM) (1 mL containing 5 × 10^4^ CCID_50_ per nostril) were intranasally instilled. After completion of inoculations, an additional aliquot of each inoculum was stored at -80 °C for estimation of actual received viral dose by viral titration.

Throughout the study, animals were visually inspected daily for clinical signs of illness and evidence of reproductive losses. On days 0, 6, 8, 10, and 14, physical examinations were performed and blood samples were collected for VI. On day 14 and then every 14 days until does gave birth, blood was collected for VN, and pregnancy examinations were performed by transrectal or transabdominal ultrasound. If ultrasonic evidence of fetal non-viability or clinical signs of abortion were observed, vaginal swabs were collected for assay by reverse transcriptase-polymerase chain reaction (RT-PCR) and VI. Animals with evidence of fetal non-viability (significant reduction of intrauterine fluid, absence of fetal heart beat, and absence of fetal movement) were administered 2 mL of dinoprost tromethamine (Lutalyse®, Zoetis Inc., Kalamazoo, MI, USA) and uterine contents were collected approximately 48 – 72 h later. Crown-rump lengths were determined on recovered fetuses to estimate gestational age of death based on a previously published equation (y = 24.42 + 0.39x) [28,29]. Fetuses were dissected to collect tissues including liver, heart, thymus, skin, brain, and placenta for VI and RT-PCR.

When kids were born, viability and ability to nurse were evaluated. Kids were individually identified by ear tag, and blood samples were collected for VI and VN on the day of birth. From all live-born kids, 16 – 25 mm^2^ skin biopsies were collected from the ear pinna using ear notch pliers and placed into tubes containing phosphate-buffered saline for antigen capture ELISA and formalin-containing tubes for immunohistochemistry. When possible, a necropsy was performed on aborted and stillborn fetuses and deceased kids, and representative tissue sections were collected for VI, RT-PCR, and immunohistochemistry. Approximately 30 days after all does had given birth, blood samples were collected from kids for VI and VN.

### Virus isolation

Detection of BVDV was performed in buffy coat cells from whole blood samples of adult goats and kids, tissues of aborted fetuses and non-viable kids, and uterine fluids through co-cultivation with MDBK cells. Briefly, the 1 mL sample suspension was layered over MDBK cells that had been seeded 24 h earlier into wells of a 6-well culture plate. Following a 1-h adsorption period, 3 mL of MEM with 10% equine serum was added. The plates were incubated for 4 days. Following a single freeze-thaw cycle to release intracellular virus, lysates from this procedure were assayed on MDBK cells in triplicate by an immunoperoxidase monolayer assay using the BVDV-specific monoclonal antibodies D89 and 20.10.6 [30].

### Virus titration

Virus titration was performed on aliquots of the inocula and sera of kids positive at birth by VI on buffy coat samples. The quantity of BVDV was determined by multiple ten-fold dilutions of samples in triplicate and employed the statistical method of Reed and Muench [31]. An immunoperoxidase monolayer assay was performed to confirm the presence of non-cytopathic BVDV as described above.

### Virus neutralization

Sera were separated from clotted blood following collection and stored at -80 °C until analysis. A standard virus neutralization microtiter assay was used for the detection and quantification of antibodies in sera of adult goats and kids. Sera were tested for neutralizing antibodies as previously described using the corresponding BVDV strain with which the group had been inoculated [32]. The antibody titer was defined as the inverse of the highest dilution with complete inhibition of staining by the immunoperoxidase test.

### Reverse transcriptase polymerase chain reaction and sequencing

Viral RNA was detected by a two-round rapid-cycle nested RT-PCR assay on vaginal swabs and uterine fluids of does with non-viable fetuses and on tissues of aborted fetuses or non-viable kids as previously described in detail [33]. If positive for BVDV, samples were purified using the QIAquick® PCR purification kit (Qiagen) according to the manufacturer’s specifications and sequenced by automated dye terminator nucleotide sequencing using both the 5’ and 3’ primers (BVD 180 and HCV 368, respectively). Consensus sequences were determined for each sample using Align X® computer software (Vector NTI Suite 7.1, InforMax, Inc., Bethesda, MD, USA) and results were used to compare the nucleotide sequences of BVDV in samples with those of BVDV 1b AU526 or BVDV 2 PA131 used for inoculation.

### Antigen capture ELISA

BVDV antigen detection was performed on skin biopsy samples of live-born kids using a commercially available kit (IDEXX Laboratories, Westbrook, ME 04092, USA) developed for BVDV detection in bovine samples, according to the manufacturer’s instructions. Samples were classified as positive if the sample to positive (S/P) ratio was ≥ 0.30.

### Immunohistochemistry

Ear skin biopsies collected at birth from live-born kids were submitted for immunohistochemical detection of BVDV antigen at the BVDV laboratory at Auburn University. The monoclonal antibody 15C5 (Syracuse Bioanalytical, East Syracuse, NY, USA) that specifically targets the BVDV glycoprotein E^rns^ was used to detect BVDV antigen as previously described [34]. Additionally, immunohistochemical analysis was also performed on tissues of suitable stillborn fetuses and deceased kids in the laboratory of one of the co-authors (BWB). Briefly, sections of formalin-fixed paraffin-embedded tissues were cut at 4 μm. Slides were deparaffinized and stained on an automated immunohistochemical stainer (Ventana Medical Systems, Inc., Tucson, AZ, USA). The monoclonal antibody 15C5 (Idexx Laboratories, Westbrook, ME, USA) was used as the primary antibody [35,36]. Positive and negative controls for BVDV staining consisted, respectively, of a slide containing known positive bovine tissue along with slides of test samples using an irrelevant primary antibody. After deparaffinization on the immunohistochemistry stainer, the slides were incubated with Protease III (Ventana Medical Systems, Inc., Tucson, AZ, USA) for 12 min. Before application of the primary antibody (optimally diluted at 1:5000), a blocking step using Antibody Diluent (Ventana Medical Systems, Inc., Tucson, AZ, USA) for 12 min was applied. Primary antibody incubation was for 44 min at 37 °C. Secondary antibody, alkaline phosphatase, and substrate were proprietary (Ventana Medical Systems, Inc., Tucson, AZ, USA). Tissues were counterstained with hematoxylin for four minutes and coverslipped with glass coverslips. The monoclonal antibody 15C5 has been successfully utilized in samples from heterologous hosts [13,34,37].

### Comparison of genomic sequences of progenitor BVDV and BVDV isolated from dams and PI kids

Viruses that were isolated from dams 50 (group 1) and 108 (group 2) during the acute phase of infection on day 6, the viruses from their kids (50a and 108a, respectively), and the BVDV 1b AU526 and BVDV 2 PA131 inoculum were subject to full genome sequencing to determine nucleotide changes that were introduced during the infection process. The 6 viral genomes were sequenced using a random primed, barcoded library technique that allowed the determination of 20 viral genomes simultaneously [38] using the Ion Torrent PGM platform (Life Technologies, Inc., Grand Island, NY, USA). Briefly, 140 μL of virus stock treated with a cocktail of nucleases was added to degrade host nucleic acids as previously described [39]. The viral RNA was purified and was used in reverse transcription (RT) reactions using primers consisting of 20 bases of known sequence (barcode) and 8 random nucleotides at the 3’ end [40]. Second strand cDNA was synthesized in the same tube using Sequenase 2.0 polymerase (Affymetrix, Inc., Santa Clara, CA, USA) as previously described [41]. The double-stranded cDNA was amplified using a primer consisting of the 20 base known sequence used to prime the RT reaction. The individual virus libraries were pooled so that all libraries were equimolar in the 300 to 400 base cDNA. The DNA was size-selected using a Pipen Prep (Sage Science, Inc., Beverly, MA, USA) and the 20 genome library was sequenced using the Ion Torrent with standard chemistries. After demultiplexing, the individual genomes were assembled using Lasergene 10 SeqMan NGen software (DNAstar, Inc., Madison, WI, USA). The viral sequences corresponding to BVDV isolates AU526, 50, 50a, PA131, 108 and 108a were submitted to GenBank and have the accession numbers KF835697 through KF835702, respectively.

## Results

### Clinical observations and virology for adult does

Prior to inoculation, VI and VN demonstrated that all does were seronegative and not BVDV-infected. Viral titration in aliquots of inocula that were frozen after inoculation of both groups demonstrated that the received viral dose of inoculation was 3.31 × 10^3^ CCID_50_/mL for BVDV AU526 and 4.26 × 10^3^ CCID_50_/mL for BVDV PA131. In the 14 days following inoculation, clinical signs of disease were not observed in either group, with exception of nasal discharge in 1 doe infected with BVDV PA131 on day 10. On days 6, 8, and 10 of the study, rectal temperatures remained below 39 °C in animals of group 1. On day 14, hyperthermia was detected in 2 does of group 1 (#28: 39.6 °C and #50: 40.1 °C). In group 2, elevated rectal temperatures were observed in individual animals on days 8 (#115: 39.4 °C), 10 (#77: 39.7 °C and #108: 39.8 °C), and 14 (#115: 40.1 °C). Inoculation resulted in viremia and seroconversion in 3/5 does in group 1 and 5/5 does in group 2 (Table 1).

**Table 1:** Virological assessment of pregnant goats following BVDV infection at approximately 25 days of gestation

	**Doe ID**	**Day**	**0**		**6**	**8**	**10**	**14**	**28**	**42**	**54**	**70**	**84**	**98**	**112**	**153**
			**VN**	**VI**	**VI**	**VI**	**VI**	**VI**	**VN**	**VN**	**VN**	**VN**	**VN**	**VN**	**VN**	**VN**
AU526	28		< 2	**-**	**-**	**-**	**-**	**-**	< 2	< 2	< 2	< 2	< 2	< 2	< 2	< 2
	50		< 2	**-**	**+**	**+**	**-**	**-**	256	1024	2048	2048	4096	4096	4096	4096
	76		< 2	**-**	**+**	**+**	**-**	**-**	128	256	1024	512	512	1024	1024	1024
	127		< 2	**-**	**+**	**-**	**-**	**-**	64	256	128	128	256	512	256	2048
	130		< 2	**-**	**-**	**-**	**-**	**-**	< 2	< 2	< 2	< 2	< 2	< 2	< 2	< 2
PA131	77		< 2	**-**	**+**	**+**	**+**	**-**	1024	2048	4096	4096	4096	4096	8192	4096
	108		< 2	**-**	**+**	**+**	**+**	**-**	1024	4096	8192	8192	8192	4096	16384	16384
	115		< 2	**-**	**+**	**+**	**-**	**-**	2048	2048	4096		2048	2048	2048	2048
	117		< 2	**-**	**+**	**-**	**-**	**-**	2048	4096	1024	2048	2048	512	1024	512
	132		< 2	**-**	**+**	**+**	**+**	**-**	1024	4096	8192	2048	4096	2048	4096	2048

VI – virus isolation, VN – virus neutralization.

On days 14 and 28, pregnancies of normal ultrasonic appearance were confirmed in all does except 1 in group 1 (doe 28), which had been included in the study without confident affirmation of pregnancy. On day 42, ultrasound examinations in 4/5 does in group 2 demonstrated evidence of pregnancy failure including reduced uterine fluid, lack of fetal heart beat, and lack of fetal movement. While pregnancies in group 1 continued to have normal ultrasonic appearance, more pronounced signs of pregnancy failure were detected in 4/5 does in group 2 on day 52. Additional ultrasound examinations on days 54 – 59 confirmed fetal non-viability in 4/5 does of group 2 resulting in administration of dinoprost tromethamine to induce expulsion of uterine contents.

### Clinical observations and virology for fetuses and live-born offspring

Following induction of abortion in 4/5 does in group 2, 5 fetuses were collected from 3 does, but uterine contents were not recovered from the fourth doe. Upon collection, fetuses appeared mummified, and fetal death was estimated to have occurred on study day 25–30 (55–60 days of gestation). BVDV could not be isolated from tissues of recovered fetuses; however, all tissues were positive for viral RNA by RT-PCR (Table 2). Sequence homology between the 5” UTR of BVDV from fetal tissues and BVDV PA131 was demonstrated in all recovered fetuses.

**Table 2:** Clinical and virological assessment of fetuses and kids from BVDV-infected goats

			**Day of birth**							**30-day follow-up**			
			**VI**	**VT**	**VN**	**ELISA**	**IHC**	**VI**	**PCR**	**VI**	**VT**		**VN**
	**ID**	**Description**	**WBC**	**Serum**			**Ear notch**	**Tissue**	**Tissue**	**WBC**	**Serum**	**Nasal Swab**	
AU526	50A	Viable PI	+	3.5 × 10^4^	< 2	+	Weak +	n	n	+	3.5 × 10^4^	2.0 × 10^5^	64
	50B	Stillborn	n	n	n	n	n	-	n	n	n	n	n
	76A	Normal	-	n	4096	-	-	n	n	**-**	n	n	1024
	127A	Brachygnathia viable	-	n	256	-	-	n	n	**-**	n	n	512
	127B	Normal	-	n	< 2	-	-	n	n	**-**	n	n	512
	130A	Normal	-	n	< 2	-	-	n	n	**-**	n	n	< 2
	130B	Normal	-	n	< 2	-	-	n	n	**-**	n	n	< 2
	130C	Normal	-	n	< 2	-	-	n	n	**-**	n	n	< 2
PA131	77A	Fetal mummy	n	n	n	n	n	-	+	n	n	n	n
	77B	Fetal mummy	n	n	n	n	n	-	+	n	n	n	n
	108A	Non-viable PI	+	2.0 × 10^5^	< 2	+	n	+	+	n	n	n	n
	115	Not recovered	n	n	n	n	n	n	n	n	n	n	n
	117A	Fetal mummy	n	n	n	n	n	-	+	n	n	n	n
	132A	Fetal mummy	n	n	n	n	n	**-**	**+**	n	n	n	n
	132B	Fetal mummy	n	n	n	n	n	**-**	**+**	n	n	n	n

IHC – immunohistochemistry, n – test not performed, VI –virus isolation, VN – virus neutralization, VT – virus titration.

Does in group 1 gave birth to 7 live kids and 1 stillborn fetus (Table 2). All live-born kids were active and viable, and were observed to nurse shortly after birth. Brachygnathia was observed in a male kid (#127A), but other congenital abnormalities were not detected. The stillborn fetus was approximately 60% smaller than its live-born twin, was hairless, and in an early stage of mummification. BVDV was not detected by VI in tissues of the stillborn fetus. In contrast, BVDV was isolated from buffy coat cells of the viable kid (50A) born to the same doe as the stillborn fetus. In ear skin biopsies of this BVDV-positive kid, BVDV antigen was detected by immunohistochemistry and ELISA. Serum from this animal did not contain detectable neutralizing antibodies to BVDV AU526. BVDV was subsequently isolated in buffy coat cells and serum collected 30 days after birth, confirming a persistent BVDV infection in kid 50A.

In group 2, only 1 doe (108) maintained the pregnancy to term and gave birth to a weak kid (108A) that was unable to stand or nurse. In contrast to other does of group 2, antibody titers of doe 108 increased during the last weeks of pregnancy (Table 1). BVDV was isolated from buffy coat cells in a blood sample collected from kid 108A at birth. BVDV antigen was detected on ear notches by ELISA and immunohistochemistry, and the kid was seronegative for BVDV 2 PA131 at birth. The animal was euthanized 12 h after birth as result of its moribund state. BVDV and viral RNA were identified in tissues collected at necropsy, and the kid was diagnosed as PI. At post-mortem examination, longitudinal sections of the femurs showed expanded metaphyseal regions and increased medullary density in metaphyseal and diaphyseal regions. A dense core of tan, mineralized bone filled the metaphysis and extended uniformly into the central medullary cavity of the diaphysis. The femoral metaphysis was expanded by regularly arranged, narrow spicules of retained primary spongiosa. The deep metaphyseal to diaphyseal regions contained retained cartilage cores layered on unremodeled osteoid trabeculae. Osteoclasts were not observed in the metaphysis. There was a paucity of bone marrow elements within narrowed intertrabecular spaces. Hepatic lobular architecture was disrupted by expanded portal tracts and centrilobular regions. Perivascular areas within both zones were prominent due to increased perivascular collagen, hematopoietic cells, and a moderate number of inflammatory cells. Extramedullary hematopoesis associated with the presence of many megakaryocytes was observed in hepatic sinusoids and splenic red pulp. A reduction in the number of cortical lymphocytes in examined lymphnodes and Peyer’s patches was detected. In summary, gross and histopathological lesions included long bone osteopetrosis with subsequent extramedullary hematopoiesis and lymphoid depletion.

### BVDV-antigen distribution in tissues of fetuses and live-born off-spring

While the state of mummification in 3 aborted fetuses of group 2 precluded immunohistochemical BVDV-antigen detection, tissue samples from the stillborn fetus of group 1, two mummified twin fetuses of group 2, and the PI kid of group 2 were suitable.

In placental tissues associated with the stillborn fetus of group 1, BVDV-antigen staining was observed in trophoblasts. The thymus of this fetus contained BVDV antigen in scattered random cells of the interlobular septae, smooth muscle cells of arterioles, lymphocytes, and rarely in thymic corpuscules. BVDV antigen was also detected in renal glomeruli and neurons of the cerebrum of the stillborn fetus. Very pale staining of uncertain significance was detected in hair follicles of examined skin tissues. BVDV antigen staining was absent in other examined organs including the gastrointestinal tract, kidneys, lungs, ovaries, and heart.

**Figure 1:**
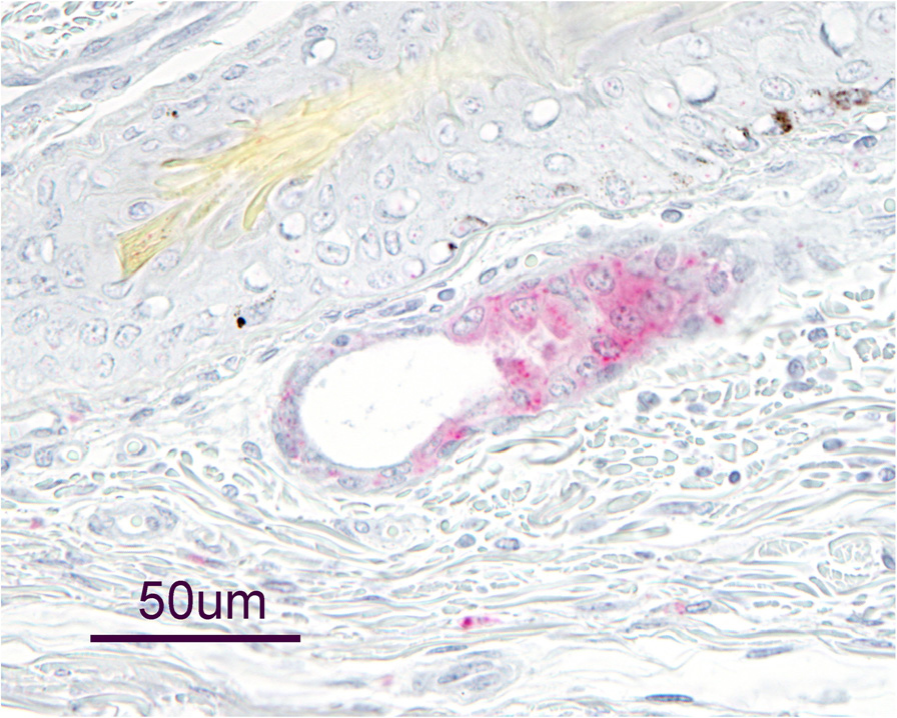
**Bovine viral diarrhea virus antigen in apocrine gland epithelium of an aborted goat fetus.** Presence of bovine viral diarrhea virus (BVDV) antigen was evaluated in tissues of a fetus aborted by a goat experimentally infected with BVDV 2 PA131 by immunohistochemistry using the monoclonal antibody 15C5. In this fetus, BVDV antigen was only detected in apocrine gland epithelium of the skin. No other analyzed tissues demonstrated positive antigen staining.

BVDV antigen was not detected in placental tissues associated with the twin fetuses recovered from doe 132 of group 2. All examined tissues of these fetuses, with exception of scattered staining in apocrine gland epithelium in the skin of one fetus (Figure 1), did not contain BVDV antigen.

**Figure 2:**
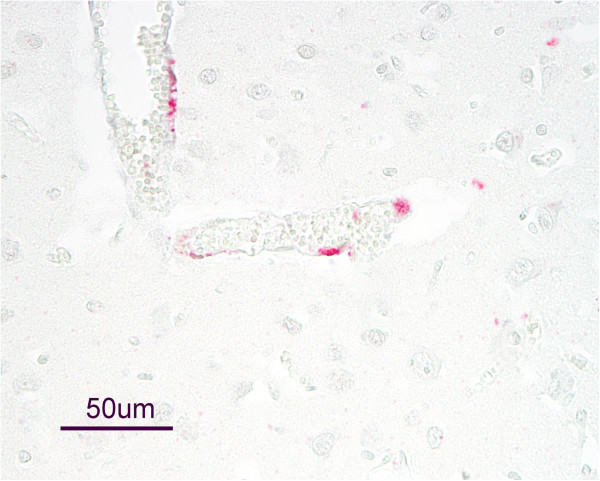
**Bovine viral diarrhea virus antigen in cerebral endothelium of a persistently infected goat kid.** A weak, non-viable goat kid was born to a goat experimentally infected with bovine viral diarrhea virus (BVDV) 2 PA131 at approximately 25 days of gestation. A diagnosis of persistent BVDV infection was made based on virus isolation and antigen detection in buffy coat cells and ear notch samples, respectively. At necropsy, BVDV was detected by virus isolation from tissues. BVDV antigen staining was present in various tissues, but most pronounced in nervous and thymic tissues.

BVDV antigen was detected in autolytic cell debris associated with trophoblasts of placental tissues of the PI kid (108A) of group 2. The distribution and intensity of antigen staining in tissues of this kid varied by organ system and was most pronounced in nervous tissues and thymus. Within the central nervous system, antigen staining was present in endothelial cells of cerebrum, cerebellum, and spinal cord (Figure 2); tunica media of the cerebrum and cerebellar meninges; neurons of the cerebrum and obex; and in optic nerve fibers near the optic cup. Antigen staining in the skin was very sparse and often limited to apocrine glands. Sparse staining was also present in epithelia of renal tubules, macrophages of the liver, epithelial cells of the exocrine pancreas, spleen, and thyroid gland. Other examined organ systems, including gastrointestinal tract, respiratory system, cardiovascular system, urogenital system, and skeletal muscles did not contain detectable BVDV antigen.

### Comparison of genomic sequences

Compared to the progenitor virus BVDV AU526 used for inoculation of group 1, 36 nucleotide changes were detected in the virus sequenced from a sample collected on day 6 from doe 50. Of these nucleotide changes, 14 (38.9%) occurred in the coding region for the non-structural BVDV proteins and 22 (61.1%) occurred in the coding region for the structural BVDV proteins. Nucleotide changes resulted in 15 amino acid substitutions, of which 8 and 7 were present in coding regions for the non-structural and structural proteins, respectively. All 36 nucleotide changes detected in its dam, were also present in the virus of PI kid 50A, in which only 2 additional nucleotide changes were detected. Of these 2, 1 was a non-synonymous nucleotide change in the coding region for the E2 protein and the other a synonymous nucleotide change in the coding region for the nonstructural protein NS4b. For group 2, inoculated with BVDV PA131, 10 nucleotide changes were detected in the virus from doe 108. Of these, 1, 4, and 5 occurred in the 5’UTR, coding region for the non-structural BVDV proteins, and coding region for the structural BVDV proteins, respectively. Of the nucleotide changes in the coding region for non-structural proteins, 3 resulted in amino acid substitutions. Also, 3 of the nucleotide changes in the structural protein coding region were non-synonymous. The genomic sequence of the virus isolated on the day of birth from PI kid 108A contained 6 of the 10 nucleotide changes detected in the virus from its dam. The remaining 4 nucleotide changes present in the dam’s virus were reverted to those of the progenitor PA131. However, as compared to the progenitor virus, 9 additional nucleotide substitutions were detected in the virus of PI kid 108A that were not present in the virus of its dam.

## Discussion

The present study evaluated the outcome of BVDV infection in pregnant goats during early gestation following experimental inoculation with BVDV 1 or BVDV 2 isolates from PI cattle. While only 3 of 5 does in group 1 became viremic and seroconverted, viremia and seroconversion was detected in all does inoculated with BVDV 2 PA131, and antibody titers in group 2 were markedly greater than in group 1. In an experimental infection study, in which seronegative heifers were challenged with either BVDV 1 or 2 at increasing doses, viremia and subsequent fetal infection occurred at dose of 10^1^ CCID_50_ of BVDV 2 but not at doses below 10^4^ CCID_50_ of BVDV 1 [42]. In this study, the received dose of viral inoculum appeared to have been approximately 1 log lower than intended for each group, possibly explaining the lower rate of viremia in group 1. However, in a study in 2-month old pigs, the utilized BVDV 1 isolate caused viremia and tissue infection at a dose that was 4 logs lower than that of BVDV 2 [43], possibly indicating that the different rates of viremia between group 1 and 2 of this study are the result of variation between individual BVDV isolates rather than differences between the 2 species of BVDV. The high rate of abortion in group 2, corresponds to previous reports of caprine BVDV infection in which reproductive failure, including fetal resorption, abortion, fetal mummification, stillbirth, and birth of congenitally infected kids with severe gross and histological pathology were the most common outcome regardless of BVDV strain [5,9,22,44]. Pregnant does infected with BVDV at various stages of gestation experienced abortions in all trimesters of pregnancy, emphasizing that BVDV should be included as a differential diagnosis for abortions in goats [5].

Clinically, the outcome of infection differed considerably between the groups. With exception of the stillborn fetus and brachygnathia in an otherwise healthy kid, infection of group 1 with BVDV 1b AU526 was clinically inapparent as is common for many BVDV outbreaks in cattle. The normal appearance and shedding of BVDV from PI kid of group 1 could have facilitated the maintenance and transmission of BVDV as has been reported in goats previously [26]. In that study, cohabitation of BVDV-naïve pregnant goats with a PI goat resulted in the birth of twin PI offspring. In contrast, cohabitation of pregnant goats with a PI calf resulted in pregnancy losses in all goats [26]. A PI goat born to a dam exposed to PI calves in Germany also appeared clinically healthy in the first months of life, but developed ill-thift, poor body condition, and anemia by 1 year of age [27]. In contrast, 2 PI goats in a recent study were born with signs of neurologic disease including tremor and ataxia [26]. Neurologic disease in that study may have resulted from infection at a later stage of gestation (38 – 45 days) as compared to this study and was reported in previous studies in fetuses and kids from goats experimentally infected at approximately 60 days of gestation [5,9].

In contrast to group 1, infection with BVDV 2 PA131 resulted in severe reproductive disease in 4 of 5 does of group 2. The only kid born in group 2 was non-viable, and was diagnosed as PI based on the presence of BVDV in buffy coat cells and tissues, presence of BVDV antigen in ear skin biopsies as detected by immunohistochemistry and ELISA, absence of serum antibodies at birth, and presence of BVDV antigen in tissues at approximately 125 days after inoculation with BVDV. Osteopetrotic changes as observed in the long bones of this kid have been previously reported in PI calves and bovine fetuses [45,46], but not in goats. A recent study evaluating the long bone morphogenesis in bovine fetuses experimentally infected with BVDV, concluded that the observed cyclic abnormal trabecular modeling of PI fetuses is secondary to reduced numbers of osteoclasts [45]. Similarly, in the PI kid of group 2 a paucity of metaphyseal osteoclast was also detected. In contrast, normal bone trabeculae and growth plates and presence of BVDV antigen in osteocytes, osteoblast, and osteoclast were detected in PI goats in a previous report [26].

The ability of both viruses to induce persistent infection in a heterologous species (white-tailed deer) was previously demonstrated [13,47]. Similar to the present study, cohabitation of pregnant white-tailed deer with a cow PI with BVDV AU526 did not result in early pregnancy losses and all does gave birth to 1 – 2 fawns of which 3 were viable and PI and 2 were stillborn [47]. In contrast, of 9 pregnant white-tailed deer co-infected with BVDV 1 BJ and BVDV 2 PA131, only 1 gave birth to a fawn PI with PA131, while the other does experienced early pregnancy losses [13]. Similar detrimental effects of BVDV 2 PA131 were not observed in previous studies in cattle, in which abortions following experimental inoculation were not observed and PI fetuses were recovered from all unvaccinated heifers approximately 75 days following challenge [48,49]. Whether the apparent difference in fetal virulence between BVDV 1 and 2 in this study was specific to the utilized isolates or reflects a differing ability of the two BVDV species to induce persistent infection in heterologous hosts is uncertain and requires further study. In previous reports of viable PI goats, BVDV 1e and 1 h were isolated, but cohabitation of pregnant goats with PI cattle infected with BVDV 2a did not result in viable offspring [5,26,27]. In contrast, experimental BVDV 2 infection of pregnant sheep resulted in the birth of viable PI lambs [50]. While both species of BVDV can infect alpacas [51,52], only the subgenotype 1b has been isolated from PI alpacas in North America and the United Kingdom [7,53-55]. The simultaneous inoculation of pregnant alpacas with two BVDV 1b isolates of cattle or alpaca origin, respectively and a BVDV 2 strain of cattle origin, resulted in birth of crias PI with 1b of cattle or alpaca origin, but not BVDV 2 [56]. Further research is necessary to elucidate the viral factors that allow survival of the fetus and establishment of persistent infection in cattle and heterologous species.

Only scattered BVDV antigen was detected in the skin of 1 of the aborted fetuses of group 2. In contrast, different tissues of the stillborn fetus of group 1 and the PI kid of group 2 contained antigen staining. Antigen staining was most pronounced in placental tissues, thymus, and nervous tissues, which is in agreement with a previous report [57]. However, in that study, the heart was also recommended as a sample suitable for detection of BVDV in aborted caprine fetuses, but heart samples did not contain antigen in the present study. Previous studies demonstrated that fetal nervous tissues are a primary target following congenital BVDV infection in cattle, sheep, and white-tailed deer [8,34,58,59]. While BVDV antigen was not detected in goat fetuses from which BVDV was isolated in a previous study [60], nervous tissues in this and another study contained widespread antigen staining in neuronal and non-neuronal cells [57]. In another report, BVDV antigen could not be detected in tissues outside of the CNS and was restricted to the brain stem and spinal cord of some fetuses from dams infected with noncytopathic or cytopathic BVDV [61]. In contrast to cattle and white-tailed deer, for which skin is a preferred sample for BVDV diagnostics and PI detection [34,62], BVDV antigen was only sparsely distributed in the skin of examined fetuses and the non-viable kid. Similarly, skin samples from only 1 of 14 aborted fetuses and neonatal goats in previous study contained BVDV antigen [57]. The ear notch sample from the PI kid of group 1 also had only weak positive antigen staining, thus suggesting that BVDV diagnostics in cases of caprine abortions and PI detection in goats should not solely rely on immunohistochemistry in skin samples.

The rapid rate of mutation that occurs during replication of BVDV is well established and has been attributed to the error-prone RNA polymerase of the virus [63]. A recent study in cattle documented that genetic change is introduced more rapidly following the establishment of single persistent infection than from multiple acute infections [64]. Similar to the present study, in which 16 (42%) amino acid substitutions were detected in the progeny BVDV AU526, in cattle, a single passage of BVDV AU526 through a PI fetus resulted in 48 nucleotide changes [64]. In pregnant cattle exposed to PI calves shedding progenitor virus, the majority of nucleotide changes detected in progeny virus from resulting PI calves were already present during the acute infection of the exposed cow [65]. In that study, a total of 48 nucleotide changes were detected between BVDV AU526 of the progenitor PI calf and BVDV AU526 from the progeny PI calf exposed in utero to the progenitor virus. Of these, 45 nucleotide changes were present in virus isolated from serum collected on day 6 during the acute infection of the dam, and only 3 additional nucleotide changes occurred in the progeny PI calf [65]. In the present study, the majority of nucleotide changes detected in the PI kid of group 1 were also present in samples collected during the acute infection of the dam. In contrast to these findings and previous reports in cattle, the number of nucleotide changes in BVDV PA131 detected following acute infection of the dam was equal to that resulting from passage in the PI fetus. The relatively large proportion of genetic change that occurred in fetus 108A and the resulting immunological response of the dam may have had a role in the high rate of abortion in group 2. The immunological response of the dam to changes in the viral genome originating from the PI fetus and the specificity of this response is currently unknown. In PI cattle, the immunological tolerance to the persistent virus is highly specific, and CD4+ T-cells from PI cattle challenged with a heterologous virus can recognize single amino acid differences between the challenge and persistent virus [66]. In pregnant heifers carrying a PI fetus, the expression of IFN-stimulated gene 15kd following BVDV infection was considerably lower than in heifers pregnant with a transiently infected fetus [67]. Furthermore, prolonged downregulation of chemokine receptor 4 and T cell receptor pathways was observed in blood of cattle pregnant with a PI fetus [68]. If the maternal immunosuppression were as specific to the infecting virus as in PI cattle, ongoing viral mutation in the infected fetus may result in immunoreactivity and failure of pregnancy. Alternatively, the greater rate of reproductive failure in group 2 may have resulted from the ability of BVDV 2 isolates to cause transplacental infections more readily, as has been suggested in previous studies [69,70].

In summary, infections of pregnant goats with BVDV 1 or 2 resulted in considerably different outcomes. While infection with BVDV 2 PA131 resulted in severe reproductive disease as is common for BVDV infection in goats, infection with BVDV 1 AU526 was clinically less apparent and could have resulted in dissemination of the virus. These results emphasize that in addition to an immediate effect on reproductive health, BVDV may have the potential for maintenance in heterologous species, including goats.

## Competing interests

The authors declare that they have no competing interests.

## Authors’ contributions

TP and PHW designed the study; TP, KPR, MAE, MFC, and PHW performed the experiments in goats; KRP, PKG, and YZ performed virological analyses; JDN performed viral whole genome sequencing; BWB performed immunohistochemical BVDV detection; HLW performed post-mortem examinations. All authors contributed to draft the paper and read and approved the final manuscript.
